# Cyclin D_1 _and Rb protein expression and their correlation with prognosis in patients with colon cancer

**DOI:** 10.1186/1477-7819-4-5

**Published:** 2006-01-20

**Authors:** Gregory Kouraklis, Stamos Theocharis, Panayiotis Vamvakas, Costas Vagianos, Andromahi Glinavou, Costas Giaginis, Crysoula Sioka

**Affiliations:** 1 Second Department of Propedeutic Surgery, Medical School University of Athens, Laiko General Hospital, Athens, Greece; 2 Laboratory of Forensic Medicine and Toxicology, Medical School University of Athens, Laiko General Hospital, Athens, Greece; 3 Department of Surgery, University of Patras, Greece

## Abstract

**Background:**

Cyclin D1 plays a major role as a potential contributor to the multistep process of oncogenesis; nevertheless its prognostic significance in colon cancer has already been examined in a few studies and needs to be further delineated. The aim of this study was to assess the expression of cyclin D1 and pRb and to correlate them with tumor histological stage and grade, proliferative capacity (Ki-67 labeling index) and clinical parameters, in order to delineate their impact on prognosis.

**Methods:**

One hundred and eleven patients, who underwent surgical resection of the colon for colon cancer constituted the group of our study. The immunohistochemical expression of cyclin D1, Rb and Ki-67 proteins was examined and correlated with clinico-pathological parameters and survival.

**Results:**

The 5-years survival rate of patients presenting cyclin D1 positive tumors was 54%, while that of cyclin D1 negative ones was 67% (P = > 0.05). The survival rate of patients with pRb positive tumors was similar to that of pRb negatine ones. Significant association was observed between Ki-67 and cyclin D1 positivity (P = 0.045). Univariate analysis revealed worse survival in advanced stage patients presenting cyclin D1 positive tumors (P = 0.025). Additionally, the survival of patients aging less than 70 years old was correlated to cyclin D1 positivity (P = 0.009). Multivariate survival analysis revealed statistical significance for stage and hepatic metastasis.

**Conclusion:**

Even though cyclin D1 and pRb have not disclosed any clear association with shorter survival, cyclin D1 positivity may be a useful predictor of subgroup patients with colon cancer being in advanced stage and early age.

## Background

In colon cancer patients, histological stage has been considered as the most important predictor of recurrence. However, for better management of patients, especially those within the same stage, additional factors should be examined. The product of the retinoblastoma gene (pRb) is the master regulator of entry into the cell- cycle in normal cells being deregulated in almost all tumors [1,2]. The pRb activity is regulated by D-type cyclins and can be downmodulated by kinase inhibitors. The discovery of cyclins and their associated catalytic subunits, the cyclin dependent kinases (cdks), as key regulators of the cell-cycle progression, opened up a new debate on their possible involvement in tumorigenesis [1-3]. Recently has been demonstrated that cyclin D1 acts as an oncogene *in vitro *and *in vivo *[4]. Once cells have entered the cell-cycle they are normally committed to go on and divide, hence the so called G1 cyclins have a predominant role in pushing cells toward progression. The cyclin D1/cdk4, cyclin D1/cdk6 and cyclin E/cdk2 complexes are the main regulators of the G1→S transition, each of them controlling a different and discrete rate limited step [2,5,6]. Among these, cyclin D1 presents the strongest evidence of a potential contribution to the multistep process of oncogenesis [7]. However, limited number of studies have examined the association of cyclin D1 expression and prognosis of patients with different malignant tumors [8-12], while others have evaluated it in colon adenocarcinoma with equivocal results [13-16]. According to the previously published studies the role of cyclin D1 remains controversial with studies to report both positive and negative prognostic values since the role of cyclin D1 is complex and multiple.

In the present study, cyclin D1 and pRb expression was assessed immunohistochemically in tumoral tissues obtained from colon adenocarcinoma patients. Cyclin D1 and pRb immunohistochemical data were correlated with tumor histological stage and grade, proliferative capacity (Ki-67 labeling index), and clinical parameters, in order to delineate their impact on prognosis.

## Patients and methods

### Patients

One hundred and eleven consecutive patients, who underwent colonic surgical resection due to colon cancer constituted the group of our study (age range, 42–94; mean 70.6 years). Patients with unresectable colon cancer were excluded. Rectal cancer was also excluded from our study as most reports suggest that the survival of rectal cancer patients is poorer than that observed in colon cancer ones. Additionally, survival rate diminishes as rectal tumors are located more distally in the bowel. Sixty six of the patients were men (59.4%) and 45 (40.6%) were women. No patient received chemotherapy or radiation therapy before surgery. Surgical treatment was curative in all patients. Adjuvant chemotherapy was given in patients when the serosa was infiltrated by the tumor. Tumors were located in the cecum and the ascending colon in 31 cases (27.9%), in the transverse in 7 (6.3%), in the descending colon in 11 (9.9%) and in the sigmoid in 62 ones (55.9%). The resected tumors were histologically staged according to Dukes classification as: A, 16 cases (14.4%); B, 44 (39.6%); C, 26 (23.4%); and D, 25 (22.5%). Three levels of differentiation were used to classify grading as: well differentiated, 8 cases (7.2%); moderately, 83 (74.8%); and poorly, 20 (18%). The patients were followed for a time interval of 2 up to 68 months, mean 38.3 ± 16.7 months, median 38 months. Patients who were died during two months after surgery were excluded from the study, avoiding with this way deaths due to postoperative complications.

### Immunohistochemistry

A mouse (IgG_1 k_) monoclonal antibody that reacts with human cyclin D1 (Santa Cruz Biochemicals, Santa Cruz, Calif., USA), a rabbit polyclonal one that reacts with human pRb (Santa Cruz Biochemicals), and another mouse (IgG_1 k_) monoclonal antibody (MIB-1) that reacts with Ki-67 antigen (Dakopatts, Glostrup, Denmark) were used in this study. Formalin-fixed paraffin-embedded tissue sections of 5 μm thickness were dewaxed in xylene and were brought to water through graded alcohols. Antigen retrieval was performed for Ki-67 antigen detection only, by microwaving slides in 10 mM citrate buffer (pH 6.0) for 15 min at high power, according to the manufacturer's instructions. To remove the endogenous peroxidase activity, sections were treated with freshly prepared 0.3% (vol/vol) hydrogen peroxide in methanol in dark, for 30 min, at room temperature (RT). Non-specific antibody binding was then blocked with a ready to use blocking reagent (Snipper, Biocare Biochemicals, Walnut Creek, CA, USA), for 10 min. The sections were then incubated for 1 h, at RT, with the primary antibodies diluted 1:100 in phosphate buffered saline (PBS). After washing three times with PBS, sections were incubated for 20 min at RT with linking biotinylated reagent, rabbit anti-mouse immunoglobulins (Dakopatts) for cyclin D1 and Ki-67 detection, and swine, anti-rabbit immunoglobulins for pRb one, both diluted 1:200 in PBS. The slides were then rinsed three times with PBS followed by incubation with peroxidase-conjugated streptavidin label (AB Complex, Dakopatts) for 20 min, at RT. Peroxidase activity was developed in 0.5% (vol/vol) 3,3'-diaminobenzidine hydrochloride (DAB, Sigma, Saint Louis, MO, USA) in PBS containing 0.03% (vol/vol) hydrogen peroxide for 2 min. Sections were counterstained with Harris' hematoxylin and mounted in gelatin (Sigma).

The stained sections were independently assessed by two different observers and specimens were considered "positive either for cyclin D1 or pRb" when more than 5% of tumor cells within the section were positively stained. Five percent was used as the cutoff value as referred in other studies [13,15,17]. The pattern of cyclin D1 and pRb immunostaining in positive cases was characterized either as nuclear only, or cytoplasmic and nuclear or cytoplasmic only. The intensity of cyclin D1 immunostaining in tumor cells was also semiquantitatively assessed as low (+), moderate (++), or high (+++). Control slides included in each experiment consisted of specific tissues previously shown to express the antigen of interest as positive controls (i.e. tonsil for cyclin D1 and pRb; normal colon for Ki-67). Internal experimental negative controls were also included by omission of the primary antibody. The fraction of Ki-67 positive cells was evaluated using a four graded scale of 0–5%, 6–25%, 26–50%, and >50%. Intraobserver discrepancies (<10%) were restained and reevaluated, and agreement was reached.

### Statistical Analysis

Statistical analysis was performed using the statistical package SPSS version 10.00. The correlation between cyclin D1, pRb and Ki-67 expression and various clinico-pathological variables (age, location, stage and grade, lymph node and liver metastasis and venous invasion) was assessed using the chi-square test. The Mann-Whitney U test was also applied in order to compare the correlation between Cyclin D1, pRB and Ki-67 expression in the colon cancer cases of our study. Survival curves were constructed using the Kaplan-Meier method and compared using the log rank test. The influence of each potential prognostic factor on the patients' survival was evaluated using Cox regression analysis. Results were considered statistically significant when P-value was less than 0.05.

## Results

Clinico-pathological characteristics of patients and tissue samples are summarized in Table 1. Positive cyclin D1 immunostaining was observed in 71 out of 111 patients (63.9%). The pattern of cyclin D1 staining was nuclear only in 16 out of 71 tumors (22.5%), cytoplasmic only in 3 out of 71 (4.2%) and cytoplasmic and nuclear in 52 out of 71 ones (73.2%). The intensity of cyclin D1 immunostaining was assessed as low in 34 out of 71 cases (47.9%), moderate in 26 out of 71 (36.6%), and high in 11 out of 71 ones (15.5%). There was no statistically significant association between cyclin D1 expression and clinico-pathological variables, as age, tumor location, histological stage or grade, lymph node or hepatic metastasis, and vessel invasion (Table 1). The pattern and intensity of cyclin D1 immunostaining was also not significantly associated with any tumor or patient characteristics (unpublished data).

**Table 1:** Cyclin D1 and pRb expression in relation to different clinico-pathological characteristics (X^2^)

**Variable**	**No of cases**	**Cyclin D1 expression**		**P value**	**pRb expression**		**P value**
		**Negative**	**Positive**		**Negative**	**Positive**	
Age							
<70	52	15	37	0,139	18	34	0.770
>70	59	25	34		22	37	
Dukes' stage							
A and B	60	22	38	0,881	22	38	0.881
C and D	51	18	33		18	33	
Differentiation							
Well + Moderate	91	36	55	0,099	30	61	0.151
Poor	20	4	16		10	10	
Tumor location							
Proximal	38	12	26	0,480	17	21	0.168
Distal	73	28	45		23	50	
Lymph node metastas							
Negative	68	22	45	0,541	23	45	0.541
Positive	43	17	26		17	26	
Liver metastasis							
Negative	93	36	57	0,182	34	59	0.794
Positive	18	4	14		6	12	
Venous invasion							
Negative	83	31	52	0,620	28	55	0.385
Positive	28	9	19		12	16	

Seventy one patients presented positive staining for pRb did not show any statistical significant association with clinico-pathological variables, as age, tumor location, histologic grade and stage, lymph node or hepatic metastasis, and vessel invasion (Table 1). The only pattern of immunostaining observed in pRb positive cases was the nuclear one. The intensity of pRb staining was assessed as low in 30 out of 71 cases (42.2%), as moderate in 20 out of 71 (28.2%), and as high in 21 out of 71 (29.6%). The survival rate of patients with pRb positive tumors was similar to that of pRb negative ones (data not shown). Among the 40 cases of pRb negative stained tumors, 18 of them were cyclin D1 negative. pRb expression was not statistically significantly associated with that of cyclin D1. The pattern of cyclin D1 immunostaining was not significantly associated with pRb expression (unpublished data).

To determine the proliferative capacity in colon cancer cases with abnormal expression of pRb and/or cyclin D1, the percentage of Ki-67 positive cells was assessed (Table 2). Intraobserver agreement was documented by Kappa statistics (K value = 0.899), that was statistically significant (P = 0.000). Statistically significant association was observed between tumor proliferative capacity expressed as Ki-67 labeling index and positive cyclin D1 staining (P = 0.045), but not with pRb one. Among the 71 tumors presenting cyclin D1 positivity, 25 assessed to have a fraction of Ki-67 positive cells of 6–25%, 12 have 26–50%, and 30 have >50%. Among the 71 patients presenting pRb positive tumors, 39 assessed to have a fraction of Ki-67 positive cells of >50%, 14 have 26–50%, and 21 have 6–25% (P = 0.217, Table 2).

**Table 2:** Immunohistochemical expression of pRb and Ki-67 in relation to cyclin D1 expression.

	**pRb**		**P value**	**Cyclin D1**		**P value**
	**pos**	**negative**		**pos**	**negative**	
Ki-67						
0–5%	6	7	0.217	4	9	0.045
6–25%	21	16		25	12	
26–50%	14	6		12	8	
>50%	30	11		30	11	

No of cases	71	40		71	40	

**Figure 1:**
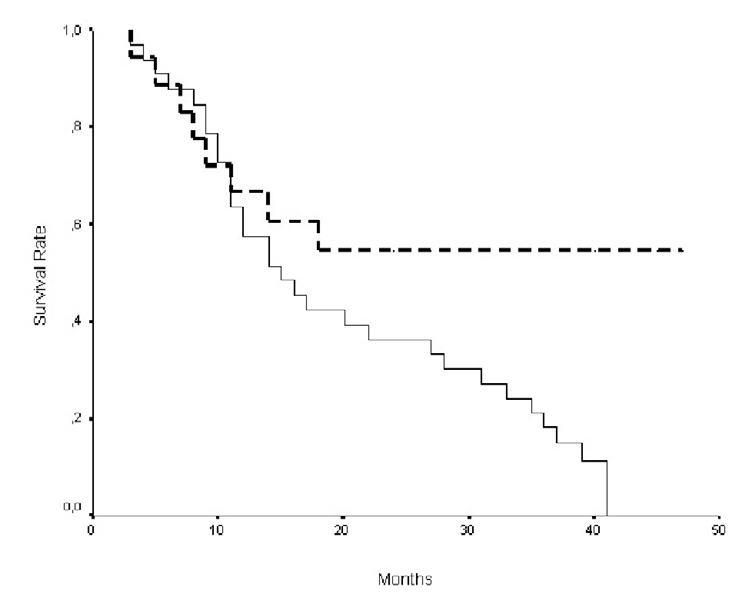
Kaplan-Meier survival curves for 51 patients in stages C and D according to cyclin D1 protein expression [protein positive (solid line), n = 33; protein negative (dotted line), n = 18; P = 0.025 by the log rank test].

**Figure 2:**
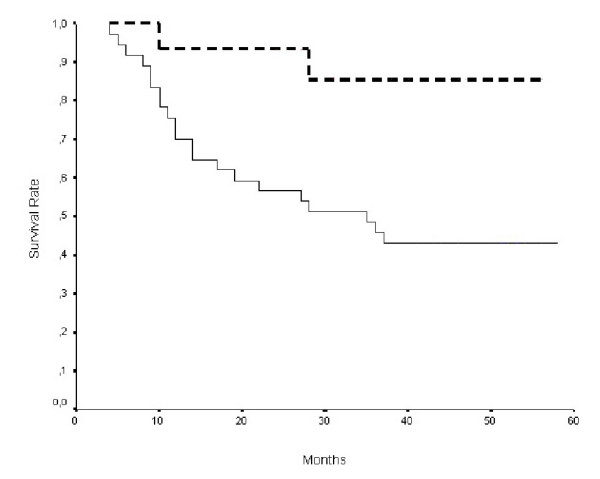
Kaplan-Meier survival curves for 52 patients <70 years old according to cyclin D1 protein expression [protein positive (solid line), n = 37; protein negative (dotted line), n = 15; P = 0.009 by the log rank test].

The 5-years survival rate of patients with cyclin D1 positive tumors was 54%, while that of patients with cyclin D1 negative ones was 67%. Even though the survival of patients with cyclin D1 positive tumors was shorter, the difference was not statistically significant. The survival of patients presenting advanced stage (stage C and D) and cyclin D1 positive tumors was significantly worse than that of patients with cyclin D1 negative ones (P = 0.025, figure 1). Additionally, the survival of patients less than 70 years old was significantly correlated to positive cyclin D1 expression (P = 0.009; figure 2). Multivariate survival analysis revealed statistical significance for stage and hepatic metastasis (Table 3).

**Table 3:** Cox proportional hazard model for factors associated with survival of colon cancer.

Variable	Relative risk	95% CI	P value
Dukes' stage			
AB vs CD	8.59	2.67–27.56	**0.003**
Grade			
Well+moderate vs. poor	1.29	0.64–2.61	0.471
Site			
Proximal vs. distal	1.00	0.52–1.92	0.989
Lymph node metastasis			
Negative vs. positive	1.41	0.55–3.62	0.471
Hepatic metastasis			
Negative vs. positive	0.12	0.05–0.31	**0.000**
Vessel invasion			
Negative vs. positive	0.81	0.40–1.65	0.569
Adjuvant therapy			
Yes vs. No	0.28	0.03–2.23	0.233
pRb			
Negative vs. positive	0.83	0.43–1.60	0.580
Cyclin D1			
Negative vs positive	1.03	0.50–2.12	0.919

CI = Confidence interval

## Discussion

In the present study, the expression of cyclin D1 and pRb in 111 cases of resected primary human colon carcinomas was investigated. The frequency of cyclin D1 positivity revealed in colon carcinoma cases was 63,9%, being similar to the results described by Arber *et al *[14], and higher than that reported by Palmqvist *et al *[13]. In other studies, neither the pattern nor the intensity of cyclin D1 staining was scored. Differences in the pattern of cyclin D1 staining were noted in the colon cancer cases examined in our study. The presence of cytoplasmic pattern could suggest that not only elevated cyclin D1 levels occurring in colon cancer tumoral cells but alterations in the transport of the protein in the nucleus also exist. Due to the variety of parameters used, (e.g. antibodies, tissue fixation, threshold definitions for positivity or overexpression, semiquantitative scales), it is often difficult to make a direct comparison between the results obtained in different studies.

We have also reported lack of correlation between cyclin D1 and pRb positivity and clinico-pathological tumor characteristics. Positive cyclin D1 and pRb stainings did not show any specificity to the location of the tumor. Our results contrast with those of other studies that showed strong specificity to the right colon [13,18], emphasizing the possibility that cancers developed in the right colon have a different spectrum of genetic damage and biological behavior than those developed in the left colon and rectum.

In our study, the proliferate activity (Ki-67 protein expression) in colon cancer cases was correlated with cyclin D1 levels (P = 0.045, Table 2) suggesting that cyclin D1 contributes to an increased proliferate potential that might be adequate in colon cancer cases [19]. On the contrary, opposite results have been reported in breast [20], and hypopharyngeal [21] cancer. Thus, cyclin D1 might be important in the mechanisms behind malignancies, and it confers growth advantage, according to the obvious function in the G1-S transition.

It has been shown that cyclin D1 expression correlated with poor survival rate in a variety of human malignancies [8-10]. Previous studies have proposed a link between cyclin D1 expression and short survival rate in colon cancer patients [13,15], while this correlation was not observed in three other published series [14,16,22,23]. In the present study, only the survival of patients with cyclin D1 positive and advanced tumor stage (stage C and D) was significantly worse (P = 0.025). Our study also revealed that the survival rate of patients aged less than 70 years old presenting cyclin D1 positivity was significantly worse (P = 0.009), suggesting possibly that environmental effects are likely to dominate over genetic ones in older patients [24]. This hypothesis has been reinforced recently by the study of Lichtenstein et al [25].

In our study of 111 colon cancer cases, the detection of pRb positivity has not disclosed a clear association with poor survival (P = 0.444). Regarding cyclin D1 positivity, our data indicate an association with shorter survival. We found a very significant correlation between Dukes' stage and poor clinical survival (P = 0.000). However, as in the clinical practice, the histological staging and differentiation provide the predominant prognostic information; it is therefore relevant to evaluate the relative contributions of cyclin D1 and pRb expression as compared to the clinico-pathological features. This evaluation can be performed by forward analysis in a Cox model [26]. In such analysis, we did not find any statistical significance, or a higher relative risk factor of poor prognosis for pRb and cyclin D1 positivity within the same confidence interval. Our results are in agreement with those of others [13,27] who also did not found any significant correlation.

The frequency of pRb positivity was 63.9%, and it was substantially more frequent than its inactivation. The frequency of pRb expression is in agreement with earlier observations that approximately one-third of colorectal cancers have increased copy numbers of one Rb1 allele [28,29], a 2- to 5- fold increase in Rb protein and mRNA levels compared with adjacent normal mucosa [30], as well as a 2-fold increase of the abundance of pRb and its phosphorylation status compared with normal mucosa [31]. It is known that the Rb gene is a tumor suppressor one and it is difficult to explain its overexpression in colon cancer. Presumably, it cannot be excluded that the Rb gene might contain genetic abnormalities which could lead to a functional inactivated protein with a subsequent longer degradation resulting in high protein levels [28,32].

In conclusion, our study did not demonstrate an overall significantly worse prognosis in patients with cyclin D1 or pRb positivity. However, it showed that the survival rate of a patients subgroup presenting in advanced stage, or in patients aged less than 70 years old with cyclin D1 positive tumors was significantly worse. Consequently, our study indicates that cyclin D1 expression may be a predictor of patients presenting in advanced stage and of patients at young age. Thus, the importance of cyclin D1 expression seems to be complex and different between tumors, and therefore, our study encourage further and larger studies for cyclin D1 expression in colon cancer, in order to verify if the expression of this molecule indicates poor prognosis.
